# Danger of Glazed Earthenware Vessels

**Published:** 1883-12

**Authors:** 


### Danger of Glazed Earthenware Vessels.

M. Perusson, a chemist at Limoges, furnishes fresh evidence of the danger of using glazed earthenware vessels, inasmuch as the glaze frequently contains lead oxide, which becomes soluble in the presence of acids. M. Perusson cites the following instance: One hundred grammes of milk was left to ferment in a glazed receptacle, and twenty-two centigrammes of lead sulphate was removed from it. When the glaze becomes rugged, the interstices are filled with metallic and fermenting substances; thus the danger is increased. Such utensils should either not be used, or else submitted to the influence of the direct contact of flame, or, in other words, singed. This is the only method to render them harmless.—*British Medical Journal.*

